# Short-course antibiotic regimen compared to conventional antibiotic treatment for gram-positive cocci infective endocarditis: randomized clinical trial (SATIE)

**DOI:** 10.1186/s12879-020-05132-1

**Published:** 2020-06-16

**Authors:** Carmen Olmos, Isidre Vilacosta, Javier López, Carmen Sáez, Manuel Anguita, Pablo Elpidio García-Granja, Cristina Sarriá, Jacobo Silva, Belén Álvarez-Álvarez, María Amparo Martínez-Monzonis, Juan Carlos Castillo, José Seijas, Amanda López-Picado, Vicente Peral, Luis Maroto, J. Alberto San Román

**Affiliations:** 1 Instituto Cardiovascular, Hospital Clínico San Carlos, Instituto de Investigación Sanitaria del Hospital Clínico San Carlos (IdSSC), Prof. Martín Lagos s/n, 28040 Madrid, Spain; 2 Servicio de Cardiología, Instituto de Ciencias del Corazón (ICICOR), CIBERCV, Valladolid, Spain; 3 Servicio de Medicina Interna-Infecciosas, Instituto de Investigación Sanitaria del Hospital Universitario de la Princesa, Madrid, Spain; 4 Servicio de Cardiología, Hospital Universitario Reina Sofía de Córdoba, Córdoba, Spain; 5 Servicio de Cirugía Cardiaca, Hospital Universitario Central de Oviedo, Oviedo, Spain; 6 Servicio de Cardiología y Unidad Coronaria, Complejo Hospitalario Universitario de Santiago de Compostela, Santiago de Compostela, Spain; 7 Unidad de Investigación y Ensayos Clinicos. Instituto de Investigación Sanitaria del Hospital Clínico San Carlos (IdSSC), Madrid, Spain; 8 Servicio de Cardiología, Hospital Universitario de Son Espases, Palma de Mallorca, Spain

**Keywords:** Infective endocarditis, Short-course, Antibiotic therapy

## Abstract

**Background:**

Most serious complications of infective endocarditis (IE) appear in the so-called “critical phase” of the disease, which represents the first days after diagnosis. The majority of patients overcoming the acute phase has a favorable outcome, yet they remain hospitalized for a long period of time mainly to complete antibiotic therapy. The major hypothesis of this trial is that in patients with clinically stable IE and adequate response to antibiotic treatment, without signs of persistent infection, periannular complications or metastatic foci, a shorter antibiotic time period would be as efficient and safe as the classic 4 to 6 weeks antibiotic regimen.

**Methods:**

Multicenter, prospective, randomized, controlled open-label, phase IV clinical trial with a non-inferiority design to evaluate the efficacy of a short course (2 weeks) of parenteral antibiotic therapy compared with conventional antibiotic therapy (4–6 weeks).

Sample: patients with IE caused by gram-positive cocci, having received at least 10 days of conventional antibiotic treatment, and at least 7 days after surgery when indicated, without clinical, analytical, microbiological or echocardiographic signs of persistent infection. Estimated sample size: 298 patients. Intervention: Control group: standard duration antibiotic therapy, (4 to 6 weeks) according to ESC guidelines recommendations. Experimental group: short-course antibiotic therapy for 2 weeks. The incidence of the primary composite endpoint of all-cause mortality, unplanned cardiac surgery, symptomatic embolisms and relapses within 6 months after the inclusion in the study will be prospectively registered and compared.

**Conclusions:**

SATIE will investigate whether a two weeks short-course of intravenous antibiotics in patients with IE caused by gram-positive cocci, without signs of persistent infection, is not inferior in safety and efficacy to conventional antibiotic treatment (4–6 weeks).

**Trial registration:**

ClinicalTrials.gov Identifier: NCT04222257 (January 7, 2020).

EudraCT 2019–003358-10.

## Background

Infective endocarditis (IE) is a serious disease with significantly high mortality rates of up to 22% in the case of in-hospital mortality, and 45% at 5 years [1]. Globally, IE is responsible for 65,000 deaths per year [1] and is burdensome in terms of resources.

The incidence of the disease has increased over time, along with development of diagnostic techniques and occurrence of significant changes in the clinical and microbiological profile of the episodes [2]. Nowadays patients diagnosed with IE are older, have much comorbidities, such as diabetes mellitus, chronic renal failure, chronic obstructive pulmonary disease, or cardiac implantable electronic devices, and a higher level of frailty compared to previous decades [3, 4].

Regarding microorganisms causing IE, the most frequently involved group is staphylococci, particularly *Staphylococcus aureus*; however its incidence has decreased over the last decade in favour of coagulase-negative staphylococci, and enterococci. This fact is most probably due to a progressively older population as well as a more frequent invasive manipulation of patients (such as the use of intravenous catheters, haemodialysis and other invasive techniques) [2, 5].

Antibiotic therapy, together with cardiac surgery when indicated, is the keystone of treatment for IE, and guidelines recommend that it should be administered intravenously and for a long time, often between four to six weeks [6]. Prolonged hospital stay is associated with occurrence of more medical complications (nosocomial and catheter-related infections), deconditioning and loss of functional capacity, pharmacological adverse effects due to prolonged antibiotic therapy, and a hampered quality of life both for patients and their families, together with a higher health care spending [1, 2, 6].

According to clinical practice guidelines for the diagnosis and treatment of IE, in order to shorten hospital admission duration therapy, programs on an outpatient setting must be developed, either at the hospital or at the patient’s home. Outpatient parenteral treatment is a feasible alternative in stable patients. However, few European hospitals have the necessary infrastructure, and this option require patients and caregivers education, well trained staff and a close clinical follow-up to monitorize therapeutic efficacy and adverse effects.

Regarding the possibility of oral antibiotics administration, a recent study (POET trial) has demonstrated the efficacy and safety of this strategy, which shorten conventional therapy duration, permitting an earlier discharge from hospital to continue antibiotic treatment on an outpatient basis, even with oral therapy [7]. In this trial the authors demonstrated that oral therapy was not inferior to conventional parenteral treatment in selected stable patients.

In addition, clinical practice guidelines point out that in certain groups of patients the administration of only 2 weeks of antibiotic treatment may be sufficient [6]. In line with this recommendation, some prospective studies and clinical trials have reported the safety and efficacy of this treatment in left-sided native valve IE caused by penicillins sensitive streptococci [8], and in right-sided IE associated with the use of parenteral drugs caused by methicillin sensitive *S. aureus* [9].

In any case, general recommendations to maintain parenteral antibiotic treatment for 4 to 6 weeks in the majority of patients with IE, are mainly based on consensus documents, retrospective studies and case records [6].

Currently, there are no randomized studies demonstrating that a prolonged parenteral treatment provides more benefits than a short treatment in patients with IE and a favorable clinical evolution after the so-called “critical phase” of the disease, which corresponds to the first days after diagnosis [7, 10–12].

Moreover, the high percentage of patients who undergo surgery could facilitate an earlier and definitive eradication of the infection that could shorten the time of antibiotic treatment [13, 14]. In addition, new and more effective antibiotics able to early tissue sterilization are now available (daptomycin, fosfomycin, ceftaroline).

Thus, in stable patients with adequate response to antibiotic treatment, without signs of persistent infection or metastatic foci such as spondylodiscitis, it is likely that a shorter antibiotic regimen would be an efficient and safe alternative, which would improve patients’ quality of life, save costs, and decrease the risk of complications related to the adverse effects of prolonged antibiotic treatment.

Additionally, in the field of infectious diseases, the most recent scientific evidence suggests that short courses of antibiotics are an effective and safe alternative in different types of infections such as community-acquired pneumonias, ventilator-associated pneumonias or urinary tract infections [15–19]. Short-term course also have multiple added advantages: lower incidence of adverse effects related to antibiotic therapy, lower risk of creating antibiotic resistance, and lower risk of *C. difficile* infection [15, 20].

## Methods/design

### Hypothesis

Our hypothesis is that a two weeks short-course of intravenous antibiotics in patients with IE caused by gram-positive cocci, without signs of persistent infection, is not inferior in safety and efficacy to conventional antibiotic treatment (4–6 weeks).

### Aims

The primary aim of our study is to compare the efficacy and safety of a short-course antibiotic therapy (two weeks) in patients with IE caused by gram-positive bacteria, with those of conventional treatment (4–6 weeks).

### Study design and setting

SATIE is a multicenter, prospective, randomized, controlled open-label, phase IV clinical trial with a non-inferiority design. Participant centers will be 7 Spanish academic hospitals (Hospital Clínico San Carlos, Hospital Clínico de Valladolid, Hospital Universitario de La Princesa de Madrid, Hospital Universitario Reina Sofía de Córdoba, Hospital Universitario Central de Oviedo, Complejo Hospitalario Universitario de Santiago de Compostela, and Hospital Universitario de Son Espases, Palma de Mallorca).

### Eligible patients

Patients with definite IE according to the modified ESC 2015 criteria [6], treated for at least 10 days of appropriate parenteral antibiotic therapy overall (according to guidelines and microbiology sensitivity testing), and at least 7 days of parenteral antibiotic therapy after valve surgery when indicated, without clinical, analytical, microbiological or echocardiographic signs of persistent infection, who fulfill the inclusion criteria and do not meet any exclusion criteria (Table 1) are eligible.

**Table 1:** Inclusion and exclusion criteria

Inclusion criteria	
I. Definite IE, according to modified ESC 2015 criteria [6], caused by gram-positive cocci (staphylococci, streptococci and enterococci), including native, prosthetic valve IE and cardiac device-related IE.	
II. 18 years old or older.	
III. Absence of fever, microbiological or analytical findings suggesting persistent infection in the last 24 h prior to randomization.	
IV. Absence of locally uncontrolled infection signs (abscess, pseudoaneurysm, fistula, enlarging vegetation) at randomization, confirmed by recent transesophageal echocardiography (performed within 48 h of randomization).	
V. Women of childbearing potential who will agree to the use of effective contraceptive methods while on antibiotic treatment.	
Exclusion criteria	
I. Patients who have received appropriate parenteral antibiotic therapy for infective endocarditis for more than 12 days.	
II. Patients not suitable to be discharged after 10 days of conventional treatment, due to clinical reasons (sequels of stroke that prevent discharge, progressive renal failure, hepatic failure, heart failure).	
III. Patients receiving chemotherapy or immunosuppressive therapy.	
IV. Pregnant or breastfeeding women.	
V. Need of prolonged antibiotic therapy due to spondylodiscitis or other septic complication.	
VI. Absence of patient’s ability or commitment to continue follow-up after being discharged from hospital.	
VII. Inability to give informed consent to participation.	
VIII. Cognitive impairment or lack of language skills needed to complete the questionnaires.	
IX. Patients who meet urgent cardiac surgery ESC criteria but are considered inoperable due to high surgical risk.	

The need for urgent surgery is not an exclusion criterion and cardiac surgery will be carried out following ESC guidelines recommendations [6].

### Endpoints

The primary endpoint is a composite of all-cause mortality, unplanned cardiac surgery, clinical embolisms, and relapses within 6 months after the inclusion.

Unplanned cardiac surgery is defined as heart surgery not planned before randomization. Surgery due to sterile pericardial effusion or hemorrhage is not included. Clinical embolisms are defined as any symptomatic embolic event that occurs after randomization. Relapse has been defined as the appearance of any positive blood culture with the same primary microorganism or the progression or reappearance of typical echocardiographic findings of IE (new appearance or enlargement of a vegetation, appearance of new periannular complications) documented by transesophageal echocardiography (TEE).

Secondary objectives include the evaluation of the economic consequences and the impact on patients’ experienced quality of life, functional performance, and complications related to prolonged hospital stay (Table 2).

**Table 2:** Secondary endpoints

Perceived quality of life. We will measure changes in the mean score of SF-12 between the time of randomization and 4 weeks after.	
Functional performance. We will measure changes the change in the mean score of short performance physical battery test (SPPB) between the time of randomization and 4 weeks after.	
Risk of clinical complications related to prolonged hospital stay (nosocomial infections, intravascular catheter-related infections) in the next 6 months after the inclusion in the study.	
Total hospital length of stay in the next 6 months after the inclusion in the study, including admissions related or not related to IE.	
Total costs related with patients’ treatment in the next 6 months after the inclusion in the study.	

### Choice of antibiotics

All patients included in the study will receive intravenous antibiotic treatment in accordance with the European Society of Cardiology guidelines recommendations [6], endorsed by the Spanish Society of Cardiology. The most frequently used antibiotics in this setting are penicillin and penicillin derivatives, glycopeptides, lipopeptides and rifampin.

The control group will receive appropriate parenteral antibiotic therapy (according to guidelines and microbiology sensitivity testing) for 4 to 6 weeks, and the experimental group will receive appropriate parenteral antibiotic therapy for 2 weeks.

### Intervention

Patients will be included in the study consecutively. We will prospectively register all patients that will not be included in the study, specifying the reason. All the information will be recorded in an electronic case record form (RedCap). Patients treated for at least 10 days of appropriate parenteral antibiotic therapy overall (according to guidelines and microbiology sensitivity testing), and at least 7 days of parenteral antibiotic therapy after valve surgery when indicated, fulfilling all inclusion criteria and who do not meet any exclusion criteria, once having signed the informed consent form, will be randomized to one of the two intervention groups:

- Experimental group: patients allocated to this group will receive a short course of antibiotic therapy for 2 weeks.
- Control group: those patients allocated to continue with standard parenteral treatment will maintain antibiotic treatment for 4 to 6 weeks.

In all operated patients, valve culture will be systematically performed; in case of positive valve culture, the day of the surgery will be considered the first day of antibiotic treatment.

Randomization will be performed with blocks of 4 and 6 patients by an automated assignment system with a ratio of 1:1. In addition, randomization will be further stratified by the need of cardiac surgery using RedCap.

Criteria for discontinuing allocated interventions include: need for starting chemotherapy or immunosuppressive therapy during intervention, patients’ inability to continue follow-up after being discharged from hospital, and emergence of any exclusion criteria, except for pregnant women once antibiotics’ administration has ended.

### Follow up

Once included in the study, and during hospital stay, patients will be daily evaluated by at least one of the investigators. After discharge, patients will be instructed to measure their temperature twice a day, and to contact their doctors if it is above 38 °C, or in case of shivering. During the 6 months period after randomization, all patients included in the study will be periodically assess by the investigator in the follow-up visits (Table 3) that will include physical examination, blood tests, blood cultures and TEE.

**Table 3:**
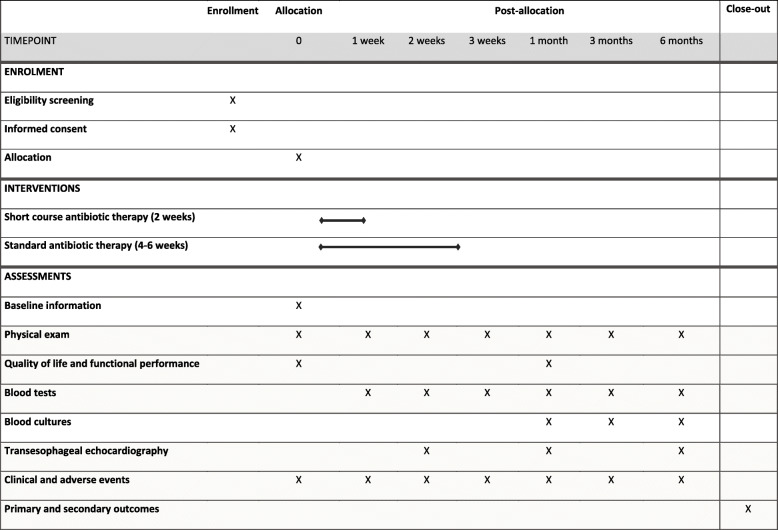
Study flow diagram

### Sample size

This study was designed as a non-inferiority trial. Most IE complications occur in the initial phase of the disease, usually in the first week. In previous studies, 6-months all-cause mortality ranged from 18 to 26% [7, 12]. Based on results and survival curves from these studies, we estimate a 2 to 5% risk of all-cause mortality after randomization. The need for unplanned surgery was estimated between 1 and 3% [12], the risk of new clinical embolic events between 1 and 2% [7], and the risk of relapse between 1 and 3% [7, 21, 22]. Thus, the risk of the primary outcome was estimated to be between 5 and 10%.

Considering a 10% composite event rate [7], selecting a non-inferiority margin of 10 percentage points, a power of 80% to confirm non-inferiority, a one-sided confidence interval of 97.5%, and assuming a 5% loss to follow-up, the number of patients to be included is 298.

### Present status

The first patient in SATIE trial will enrolled in in May 2020. The end of enrollment is projected for September 2021.

### Statistical analysis

Categorical variables will be expressed as a frequency and a percentage, and compared with the chi-square test and the Fisher exact test when necessary. Quantitative variables will be expressed as median and interquartile range or mean and standard deviation. Assessment of normality and equality of variances for continuous data will be performed using the Shapiro-Wilk test and the Levene test, respectively. Thereafter, continuous variables will be compared by a Student t-test, Mann-Whitney U test or Fisher-Pitman permutation test as necessary. First, baseline variables in both groups (control and experimental) will be compared by a chi-square test or Student t-test. If any of the variables is found to be asymmetrically distributed, the effect of the variable will be adjusted in each of the endpoints.

Endpoint analysis: Primary endpoint: differences in the primary outcome will be assessed by the evaluation of absolute and relative risk differences between both groups, calculating the confidence interval (CI) of the difference to assess non inferiority. To evaluate the impact of the intervention in the risk of each of the events included in the composite endpoint, the relative risk with 95% CI will be used, adjusted or not for potential confounding variables asymmetrically distributed, as previously expressed. Alternatively, we will also evaluate differences considering time, calculating the CI of the differences in survival free from the composite endpoint by means of Kaplan Meier curves. Secondary endpoints. 1st: to compare the mean change in the score of SF-12 questionnaire for perceived quality of life between the time of randomization and 4 weeks later, a MANOVA analysis will be used; 2nd: to compare the mean change in the score of the SPPB for functional performance between the time of randomization and 4 weeks after, a MANOVA analysis will be used; 3rd: to compare the median of total hospital stay six months after inclusion a non-parametric test will be used; 4rd: to compare the incidence of clinical complications related to prolonged hospital stay during the six months after inclusion, the risk ratio with its 95% CI will be used.

All analyses will be performed by intention-to-treat and by per-protocol. Stratified analyses of primary and secondary endpoints by the following pre-specified subgroups will be performed: age, sex, diabetes mellitus, chronic renal failure, native vs prosthetic valve IE, surgery vs medical treatment, and microorganisms (streptococci, enterococci, coagulase-negative staphylococci, *Staphylococcus aureus*).

All tests will be two-sided, and differences will be considered statistically significant at *P*-values < 0.05. Statistical analysis was performed with Stata V.12.0 (StataCorp, College Station, Texas, USA).

### Ethics

This study will follow the current legislation regarding adequate clinical practice, biomedical research, development of clinical trials, personal data protection, and will adopt the ethical principles of the Helsinki Declaration regarding human experimentation (Fortaleza, Brasil, 2013). The study protocol has been approved by the local Ethics Committee and the Spanish Agency of Medicine and Medical devices (AEMPS).

Patients will be asked to read, accept and sign an informed consent form before any information was collected. The refusal or withdraw of this consent will prevent the inclusion or continuity of the patient in the study. All documents regarding patients participating in the study will be identified with the name of the clinical trial.

### Monitoring

An independent data monitoring committee will periodically assess the progress, safety data and critical efficacy endpoints of the study. As all drugs and procedures used in the study are already approved and commercialized, the monitoring plan will be the one of a low-intervention clinical trial.

## Discussion

Infective endocarditis entails a dreadful prognosis (currently one out of five patients die in Spain during the acute phase of the disease) [2], but also carries a high burden of morbidity and disability. This disease is not only associated with clinical events that cause physical sequelae, such as strokes due to central nervous system embolisms, but is also related to prolonged admissions due to the need for long-term intravenous antibiotics (at least 4 weeks with the current recommendations), which in all cases, but especially in older patients, leads to loss of functional capacity and poorer quality of life.

It is also known that prolonged parenteral antibiotic therapy is associated with an increased risk of adverse effects and antibiotic resistance, as well as an increased risk of catheter related and *C. difficile* infections [18].

The most recent scientific evidence has shown that short courses of antibiotics are an effective and safe alternative in urinary tract infections and pneumonias, both community acquired and ventilator-associated [18–22]. We are aware though, that the evidence is little in patients with bacteremia.

With our study we intend to evaluate not only the efficacy of the shorter antibiotic regimen but whether this regimen, which allows early discharge, is associated with an improvement in patients’ quality of life and functional recovery at home, with the potential economic, health and social impact that this alternative could have.

### Limitations

Despite being a randomized clinical trial, the design is open-label. In any case, the variables included in the composite endpoint (death, relapse, unplanned cardiac surgery, symptomatic embolisms) can be objectively measured, reducing the risk of information bias. In addition, in order to minimize biases, investigators recruiting patients will be blinded to the randomized sequence, to avoid the possibility of patients selection for an specific arm, and the evaluation of adverse events will be supervised by an independent committee that will not know which group the patient belongs to.

The sample size may not guarantee the statistic power necessary to detect statistical significant differences in some of the secondary endpoints.

Randomization may not prevent the presence of confounding factors in variables that may not be measured, may not be equally distributed between the two groups, or may have a small marginal distribution but significant impact in the measured effects. External validation may be compromised, as the study will be carried out in reference centres for the treatment of IE, and due to the restrictions of the inclusion and exclusion criteria. The study will not be able to analyse potential clinical events of interest that occur beyond 6 months.

## Conclusions

SATIE will investigate whether a two weeks short-course of intravenous antibiotics in patients with IE caused by gram-positive cocci, without signs of persistent infection, is not inferior in safety and efficacy to conventional antibiotic treatment (4–6 weeks).

## Data Availability

Data sharing is not applicable to this article as no datasets were generated or analysed during the current study (study protocol).

## Abbreviations

CI: Confidence interval
ESC: European Society of Cardiology
IE: Infective endocarditis
SATIE: Short-course Antibiotic regimen compared to conventional antibiotic Treatment for gram-positive cocci Infective Endocarditis
TEE: Transesophageal echocardiography
